# Coreference annotation and resolution in the Colorado Richly Annotated Full Text (CRAFT) corpus of biomedical journal articles

**DOI:** 10.1186/s12859-017-1775-9

**Published:** 2017-08-17

**Authors:** K. Bretonnel Cohen, Arrick Lanfranchi, Miji Joo-young Choi, Michael Bada, William A. Baumgartner, Natalya Panteleyeva, Karin Verspoor, Martha Palmer, Lawrence E. Hunter

**Affiliations:** 1 Computational Bioscience Program, University of Colorado School of Medicine, Denver, CO USA; 2 Department of Linguistics, University of Colorado at Boulder, Boulder, Colorado USA; 3 School of Computing and Information Systems, The University of Melbourne, Melbourne, Australia

**Keywords:** Coreference, Annotation, Corpus, Benchmarking, Anaphora, Resolution

## Abstract

**Background:**

Coreference resolution is the task of finding strings in text that have the same referent as other strings. Failures of coreference resolution are a common cause of false negatives in information extraction from the scientific literature. In order to better understand the nature of the phenomenon of coreference in biomedical publications and to increase performance on the task, we annotated the Colorado Richly Annotated Full Text (CRAFT) corpus with coreference relations.

**Results:**

The corpus was manually annotated with coreference relations, including identity and appositives for all coreferring base noun phrases. The OntoNotes annotation guidelines, with minor adaptations, were used. Interannotator agreement ranges from 0.480 (entity-based CEAF) to 0.858 (Class-B3), depending on the metric that is used to assess it. The resulting corpus adds nearly 30,000 annotations to the previous release of the CRAFT corpus. Differences from related projects include a much broader definition of markables, connection to extensive annotation of several domain-relevant semantic classes, and connection to complete syntactic annotation. Tool performance was benchmarked on the data. A publicly available out-of-the-box, general-domain coreference resolution system achieved an F-measure of 0.14 (B3), while a simple domain-adapted rule-based system achieved an F-measure of 0.42. An ensemble of the two reached F of 0.46. Following the IDENTITY chains in the data would add 106,263 additional named entities in the full 97-paper corpus, for an increase of 76% percent in the semantic classes of the eight ontologies that have been annotated in earlier versions of the CRAFT corpus.

**Conclusions:**

The project produced a large data set for further investigation of coreference and coreference resolution in the scientific literature. The work raised issues in the phenomenon of reference in this domain and genre, and the paper proposes that many mentions that would be considered generic in the general domain are not generic in the biomedical domain due to their referents to specific classes in domain-specific ontologies. The comparison of the performance of a publicly available and well-understood coreference resolution system with a domain-adapted system produced results that are consistent with the notion that the requirements for successful coreference resolution in this genre are quite different from those of the general domain, and also suggest that the baseline performance difference is quite large.

## Background

### Context and motivation

Coreference, broadly construed, is the phenomenon of multiple expressions within a natural language text referring to the same entity or event. (By *natural language,* we mean human language, as contrasted with computer languages). Coreference has long been a topic of interest in philosophy [1–3], linguistics, and natural language processing. We use the term *coreference* to refer to a broad range of phenomena, including identity, pronominal anaphora, and apposition. Mitkov defines *cohesion* as “a phenomenon accounting for the observation (and assumption) that what people try to communicate in spoken or written form…is a coherent whole, rather than a collection of isolated or unrelated sentences, phrases, or words” [4]. As quoted by [4], Halliday and Hasan [5] define the phenomenon of *anaphora* as “cohesion which points back to some previous item.” Such cohesion is typically referred to as *anaphoric* when it involves either pronouns (defined by [6] as “the closed set of items which can be used to substitute for a noun phrase”) or noun phrases or events that are semantically unspecified, i.e. do not refer clearly to a specific individual in some model of the world. When cohesion involves reference with more fully specified nominals or events, the cohesion phenomenon is often referred to as *coreference.* The boundaries are fuzzy and not widely agreed upon, and as mentioned above, we take a very inclusive view of coreferential phenomena here.

Although it is of interest to many fields, we focus here on the significance of coreference and coreference resolution for natural language processing. In addition to its intrinsic interest, coreference resolution is important from an application point of view because failure to handle coreference is an oft-cited cause of performance problems in higher-level tasks such as information extraction [7, 8], recognizing textual entailment [9], image labeling [10], responding to consumer health questions [11], and summarization of research papers [12]. We briefly review some of those issues here. In particular, we review a body of literature that suggests that coreference and coreference resolution are important for the tasks of information extraction and recognizing textual entailment. We then review literature that suggests that coreference resolution approaches from other domains do not necessarily transfer well to the biomedical domain.

Relevant work in the areas of information extraction and event extraction abounds. Nédellec et al. reported a large performance difference on extracting relations between genes in the LLL task when there was no coreferential phenomenon involved (*F* = 52.6) as compared to when there were coreferential phenomena involved (*F*=24.4) [13]. El Zant describes the essential contribution of coreference resolution to processing epidemiological dispatches [14]. Yoshiwaka et al. found that coreference resolution improves event-argument relation extraction [15]. Kilicoglu and Bergler noted improvement in biological event extraction with coreference resolution [16]. Coreference resolution was shown to improve EventMiner event extraction by up to 3.4 points of F-measure [17]. Bossy et al. found that lack of coreference resolution adversely impacted even the best systems on the bacteria biotope task [18], and Lavergne et al. obtained better performance than the best BioNLP-ST 2011 participants on the task of finding relations between bacteria and their locations by incorporating coreference resolution into their system [19].

Similarly, the field of recognizing textual entailment [9] has quickly recognized the importance of handling coreferential phenomena. De Marneffe et al. argue that filtering non-coreferential events is critical to finding contradictions in the RTE task [20]. A review of approaches to recognizing textual entailment by Bentivogli et al. included ablation studies showing that coreference resolution affects F-measure in this task [21].

Coreference resolution is an important task in language processing in general and biomedical language processing in particular, but there is evidence that coreference resolution methods developed for other domains do not transfer well to the biological domain [22]. Kim et al. carried out an analysis of general domain coreference resolution and the various approaches to biological domain coreference resolution in the BioNLP 2011 Shared Task. They found that the best-performing system in that shared task achieved an F-measure of 0.34, lagging behind the 0.50 to 0.66 F-measures achieved on similar tasks in the newswire domain [23].

Choi et al. [24] investigated potential causes of these performance differences. They found that there were a number of proximate causes, most of which in the end were related to the lack of any ability to apply domain knowledge. In particular, the inability to recognize membership of referents to domain-relevant semantic classes was a major hindrance. For example, in a sentence like *Furthermore, the phosphorylation status of TRAF2 had significant effects on the ability of the protein to bind to CD40, as evidenced by our observations* [25], the antecedent of *the protein* is *TRAF2.* Domain adaptation by gene mention recognition (as defined in [26]) and domain-specific simple semantic class labelling of noun phrases (as described in [27]) allow a domain-adapted coreference resolution system to bring domain knowledge to bear on the problem. In contrast, a typical coreference resolution system’s bias towards the closest leftward noun group will tend to label *the ability* or *significant effects* as the antecedent, rather than *TRAF2.* We return to this point in the benchmarking section.

The general conclusion from these demonstrations of the importance of coreference resolution in natural language processing, as well as the current shortcomings in performance in coreference resolution in the biomedical literature, underline the necessity for advancements in the state of the art. Studies of coreference benefit from the availability of corpora, or bodies of natural language annotated with reference to the phenomena that they contain. For that reason, the Colorado Richly Annotated Full Text (CRAFT) corpus was annotated with all coreferential phenomena of identity and apposition. (See below for a detailed description of CRAFT). This paper describes the materials, the annotation process, the results of the project, and some baseline performance measures of two coreference resolution systems on this material.

As will be apparent from the review of related literature, the CRAFT coreference annotation differs from related projects in a number of ways. These include at least the following.

- The CRAFT project has an unrestricted definition of markable. (Following a tradition in natural language processing and corpus linguistics going back to the MUC-7 guidelines, we refer to things in a text that can participate in a coreferential relationship as *markables.* [33]) Most biomedical coreference annotation efforts have annotated only a limited range of semantic classes, [28] being the only exception to this of which we are aware. In contrast, in CRAFT, all nouns and events were treated as markables.
- The coreference annotations in CRAFT exist in connection with an extensive set of annotations of a variety of domain-relevant semantic classes. Markables are not restricted to these semantic classes, nor are they necessarily aligned with the borders of mentions of those semantic classes, but the associations open the way to investigation of the relationships between semantic class and coreference at an unprecedented scale.
- The coreference annotations in CRAFT exist in connection with complete phrase structure annotation. Again, the markables are not necessarily aligned with the borders of these syntactic annotations, but they are completely alignable.

### Related work

There is an enormous body of literature on coreferential phenomena, coreference corpus annotation, and coreference resolution in the linguistics and natural language processing literature. We can only barely touch on it here, although we try to give comprehensive coverage of the relevant literature in the biomedical domain. Panini discussed the topic, perhaps as early as the 4th century BCE [29]. The Stoics made use of the concept of anaphora [1]. The earliest references that we have found in the late modern period date to 1968 [30, 31], but there are also discussions as early as the beginning of the 20th century [32].

For comparison with the biomedical coreference annotation projects discussed below, we review here some general-domain coreference corpora:

- The MUC-6 and MUC-7 [33] Message Understanding Conferences inaugurated the modern study of coreference resolution by computers. It introduced the evaluation of coreference resolution systems on a community-consensus corpus annotated with respect to community-consensus guidelines. MUC-7 first defined the IDENTITY relation, which was defined as symmetrical and transitive. The markables were nouns, noun phrases, and pronouns. Zero pronouns were explicitly excluded. (*Zero pronominal anaphora* occurs when there is no noun or pronoun expressed, but there is understood to have been an implicit one. This is a somewhat marginal phenomenon in English, where it is often analyzable in other ways, but is quite pervasive in some languages [4]). The final MUC-7 corpus contained sixty documents.
- Poesio [34] used a corpus constructed of labelled definite descriptions to provide empirical data about definite description use. (A *definite description* makes reference to “a specific, identifiable entity (or class of entities)…identifiable not only by their name but by a description which is sufficiently detailed to enable that referent to be distinguished from all others” [6]). A surprising finding of the study with implications for the evaluation of coreference resolution systems (and for linguistic theory) that target definite noun phrases was that an astounding number of definite noun phrases in the corpus were discourse-new. The standard assumption is that noun phrases can be referred to with a definite article only when they have been previously mentioned in the discourse (modulo phenomena like frame-licensed definites, e.g. *the author* in *I read a really good book last night. The author was Dutch* [35]), so it is quite surprising that at least 48% of the 1412 definite noun phrases in their corpus did not have antecedents (defined by [6] as “a linguistic unit from which another unit in the [text] derives its interpretation”). One consequence for coreference resolution work is that it becomes very important in evaluating systems that resolve definite noun phrases (as a number of them do) to be aware of whether the evaluation includes all definite noun phrases, or only ones manually determined to actually have antecedents. If the intent is to build the former, then it becomes important for systems to have the option of returning no antecedent for definites.
- The OntoNotes project comprises a number of different annotations of the same text, in different annotation levels. These levels include coreference. The OntoNotes coreference annotation differs from most prior projects in that it includes event coreference, which allows verbs to be markables [36]. The OntoNotes guidelines were the primary source of the CRAFT coreference annotation guidelines, and OntoNotes will be discussed in more detail below. Version 4.0 of the OntoNotes data was distributed in the context of the CoNLL 2011 shared task on coreference resolution [37].

The significance of the work reported here comes in part from its focus on biomedical literature, as opposed to the large body of previous work on general-domain materials. As discussed elsewhere in this paper, general-domain coreference resolution systems have been found to not work well on biomedical scientific publications [22, 23]. This observation holds within a context of widespread differences between biomedical and general-domain text. Biomedical scientific publications have very different properties from newswire text on many linguistic levels, and specifically on many levels with relevance to natural language processing and text mining. Lippincott et al. [38] looked at similarities and differences in a number of linguistic levels of a wide variety of linguistic levels of newswire text and of scientific text in a broad cross-section of biomedical domains, and found that newswire text almost always clustered differently from scientific texts with respect to all linguistic features, including at the morphological level (e.g. distribution of lexical categories [39], marking of word-internal structure [40], relationships between typographic features and lexical category [41, 42], and sensitivity to small differences in tokenization strategies [43]), the lexical level (e.g. distributional properties of the lexicon [44], weaker predictive power of deterministic features for named entity classes [45], and length distributions of named entities [26, 46, 47]), the syntactic level (e.g. syntactic structures that are outside of the grammar of newswire text [48–50], differences in the distribution of syntactic alternations such as transitivity and intransitivity [51, 52], and longer, more complex sentences [53–55], distribution of demonstrative noun phrases [55], longer dependency chains [56], and noun phrase length and presumably complexity [55]), and the semantic level (e.g. the types and complexity of semantic classes and their relations [53], domain-specific patterns of polysemy [57], lower discriminative power of lexical features in relation encoding [58], pronoun number and gender distribution (and therefore relative usefulness or lack thereof of number and gender cues in anaphora resolution) [55, 59], distribution of anaphoric relation types [60], and prevalence of named entities versus complex noun phrases as the antecedents of anaphora [59]). Striking differences in the use of cognitively salient terms related to sensory experience and time have been noted between newswire and scientific text, as well [61]. In light of these numerous differences between newswire text and biomedical text at every linguistic level, the differences that have been noted between newswire text and biomedical text are not surprising. They motivate the work described in this paper.

We turn here to the description of a number of biomedical coreference corpora. Almost none of these are publicly available, making the significance of the CRAFT coreference annotation project clear.

- Castaño et al. [62] annotated sortal and pronominal anaphora in 100 PubMed/MEDLINE abstracts, finding that about 60% of the anaphora were sortal (meaning, in this context, roughly anaphora that refer back to an antecedent by using the category to which they belong, e.g. *MAPKK and MAPK…these kinases*).
- Yang et al. [28] annotated a corpus of 200 PubMed/MEDLINE abstracts from the GENIA data set. They demonstrated that it is possible to annotate all coreference in scientific publications. Descriptive statistics on the annotations are given in Table 1 for comparison with the distribution of annotations in the CRAFT coreference corpus.

**Table 1:** Descriptive statistics of Yang et al.’s coreference corpus [28]

	Total number	Percentage
*Anaphoric markable*		
Noun phrase	3561	29.1%
Pronoun	131	1%
*Non-anaphoric markable*		
Noun phrase	8272	67.6%
Pronoun	259	2.1%
- Kim and Park [63] created a corpus annotated with pronouns, anaphoric noun phrases with determiners, and zero pronouns. The descriptive statistics are given in Table 2.

**Table 2:** Descriptive statistics of Kim and Park’s coreference corpus [63]

Anaphoric expression	Count
Pronouns	53
Noun phrase with determiner	26
Zero anaphora	8
- Sanchez et al. [64] annotated a corpus consisting of mixed abstracts and full-text journal articles from the MEDSTRACT corpus [65] and the Journal of Biological Chemistry. A number of interesting findings came from the analysis of this corpus, including that 5% of protein-protein interaction assertions contain anaphors, with pronominal anaphors outnumbering sortal anaphors by 18 to 2, even though sortal anaphora are more frequent than pronominal anaphora in biomedical texts in general. It was also found that pleonastic *it* (the semanticsless *it* in constructions like *it seems to be the case that…*) was as frequent as referential *it* (that is, instances of *it* that do refer back to some meaningful entity in the text).
- Gasperin et al. [66] describe a biomedical coreference annotation project that was unique in a number of respects. First of all, it dealt with full-text journal articles. Secondly, the project dealt only with anaphoric reference to entities typed according to the Sequence Ontology [67]. Finally, it dealt with a number of types of bridging or associative phenomena (in which markables have a relationship other than coreferential identity). This included relations between genes and proteins, between homologs, and between sets and their members. Inter-annotator agreement statistics are given in Tables 3 and 4, calculated as kappa.

**Table 3:** Gasperin et al.’s inter-annotator agreement scores for six papers, calculated as Kappa, before and after annotation revision

	Before revision	After revision
Paper 1	0.75	0.85
Paper 2	0.70	0.83
Paper 3	0.68	0.93
Paper 4	0.62	0.95
Paper 5	0.41	0.91

**Table 4:** Gasperin et al.’s inter-annotator agreement scores for five semantic classes of anaphora, calculated as Kappa

Class/Paper	1	2	3	4	5
Coreferent	0.84	0.84	0.98	0.97	0.93
Biotype	0.84	0.81	0.92	0.88	0.79
Homolog	0.77	N/A	1.0 /	N/A	0.53
Set-member	0.78	0.69	0.66	0.83	0.88
Discourse-new	0.89	1.0	0.56	1.0	0.98
- Vlachos et al. [68] used a very semantic-class-specific annotation scheme, as in the Gasperin et al. work described above, to mark up two full-text articles from PubMed Central. They annotated 334 anaphoric expressions, of which 90 were anaphoric definite descriptions and 244 were proper nouns. Pronominal anaphors and anaphors outside of the semantic classes of interest were not annotated.
- Lin et al. [69] built a corpus consisting of a subset of MEDSTRACT [65] and an additional 103 PubMed/MEDLINE abstracts. Like Gasperin et al., they only annotated anaphoric reference to a predefined set of biologically relevant semantic classes. In all, they marked up 203 pronominal anaphors and 57 pairs involving sortal anaphors.
- Nguyen et al. [70] describe the corpus prepared for the BioNLP-ST 2011 shared task on coreference resolution. It was made by downsampling the MedCO coreference corpus described in [71] to include just those anaphoric expressions with a protein as an antecedent. The corpus was unusual in that it included relative pronouns/adjectives (e.g. *that, which, whose*) and appositives (defined below). The descriptive statistics of the resulting subcorpus are given in Table 5.

**Table 5:** Descriptive statistics of the BioNLP-ST 2011 coreference corpus [70], downsampled from [71]

	Training	Devtest	Test
Relative	1193	254	349
Pronoun	738	149	269
Definite or demonstrative	296	58	91
Noun phrase			
Appositive	9	1	3
Other	11	1	2
Antecedent	2116	451	674
Total	2247	463	714
- Chaimongkol et al. [72] differs quite a bit from other work described here with respect to the analysis of the corpus. The corpus from the SemEval 2010 Task 5 [73] was the starting data set. This data set contains articles from a variety of scientific fields. The abstracts of those articles were annotated with an extension of the MUC-6 annotation guidelines. Relative pronouns, such as *which* and *that,* were considered to be markables. The resulting corpus contains 4228 mentions and 1362 coreference chains (sets of coreferring noun phrases), with an average chain length of 3.1 mentions.
  The authors did an unusual analysis of their corpus in terms of the resolution class analysis described in [74]. They looked at the distributions of nine different types of coreferential relations in the corpus of scientific journal articles and in a number of general domain corpora, concluding that the distributions were quite different, and that scientific corpora differ from general domain corpora quite a bit in terms of coreferential phenomena. Extensive details are given in [72]. To our knowledge, this type of analysis has not been repeated with any other scientific corpora, and it appears to be a fruitful avenue for future research.
- Savova et al. [75, 76] give detailed descriptions of an annotation project that was unusual in that it used clinical data for the corpus. This corpus is also unusual in that it is publicly available. Table 6 gives descriptive statistics of the corpus, downsampled from the extensive data in [76]. Savova et al. [75] gives a very detailed assessment of the inter-annotator agreement, which was 0.66 on the Mayo portion of the corpus, and 0.41 on the University of Pittsburgh Medical Center portion of the corpus.

**Table 6:** Descriptive statistics of the i2b2 clinical coreference corpus [75, 76]

Markables	7214
Average markables per report	40.08
Pairs	5992
Average pairs per report	33.29
Identity chains	1304
Average identity chains per report	7.24

Adapted from [76]

### Summary of related work and relation to the CRAFT coreference annotation

As can be seen from the review of related literature, the CRAFT coreference annotation differs from related projects in a number of ways. The CRAFT corpus’s unrestricted definition of markable, connection to an extensive set of annotations of domain-relevant semantic classes (without restriction to those classes), and connection with complete phrase structure annotation are qualitative differences from prior work on coreference annotation in the biomedical domain. These characteristics bring biomedical coreference annotation to a scale and structure similar to that of general domain/newswire coreference annotation corpora, and should enable large steps forward both in the development of applications for coreference resolution in biomedical text and in the development and testing of theories of coreference in natural language.

## Methods

### Data

The contents of the CRAFT corpus have been described extensively elsewhere [77–80]. We focus here on descriptive statistics that are specifically relevant to the coreference annotation. Characteristics of the first version of the CRAFT Corpus that are particularly relevant to the work reported here are that it is a collection of 97 full-length, open-access biomedical journal articles that have been extensively manually annotated to serve as a gold-standard research resource for the biomedical natural language processing community. The initial public release includes over 100,000 annotations of concepts represented in nine prominent biomedical ontologies (including types of chemical entities, roles, and processes; genes, gene products, and other biological sequences; entities with molecular functionalities; cells and subcellular components; organisms; and biological processes) as well as complete markup of numerous other types of annotation, including formatting, document sectioning, and syntax (specifically, sentence segmentation, tokenization, part-of-speech tagging, and treebanking). One of the main strengths of the coreference annotation presented here is the fact that it has been performed on a corpus that has already been so richly annotated.

#### Sampling

The sampling method was based on the goal of ensuring biological relevance. In particular, the sample population was all journal articles that had been used by the Mouse Genome Informatics group as evidence for at least one Gene Ontology or Mouse Phenotype Ontology “annotation,” in the sense in which that term is used in the model organism database community. In the model organism database community, it refers to the process of mapping genes or gene products to concepts in an ontology, e.g. of biological processes or molecular functions–see [12] for the inter- acting roles of model organism database curation and natural language processing.

#### Inclusion criteria

Of the articles in the sample population, those that met unrestrictive licensing terms were included. The criteria were that they be (1) available in PubMed Central under an Open Access license, and (b) available in the form of Open Access XML. 97 documents in the sample population met these criteria.

#### Exclusion criteria

There were no exclusion criteria, other than failure to meet the inclusion criteria. All documents that met the inclusion criteria were included in the corpus.

All of those 97 articles were annotated. The current public release contains the 67 articles of the initial CRAFT release set, with the rest being held back for a shared task.

### Annotation model

#### Annotation guidelines: selection, rather than development

Recognizing the importance of the interoperability of linguistic resources [81–84], a major goal of the CRAFT coreference annotation project was to use pre-existing guidelines to the greatest extent possible. To that end, the OntoNotes coreference annotation guidelines [36] were selected. They were adopted with only one major change that we are aware of. (We should note that copyright permissions do not permit distribution of OntoNote’s guidelines (by us) with the corpus data, but the paper cited above gives a good overview of them, and the major points are described in this paper in some detail. More details are available in [77]. Furthermore, copies of the full guidelines can be obtained directly from the OntoNotes organization).

#### OntoNotes

OntoNotes [85] is a large, multi-center project to create a multi-lingual, multi-genre corpus annotated at a variety of linguistic levels, including coreference [36]. As part of the OntoNotes project, the BBN Corporation prepared a set of coreference annotation guidelines.

### Annotation guidelines

**Markables in the OntoNotes guidelines** Per the OntoNotes guidelines, markables in the CRAFT corpus include:

- Events
- Pronominal anaphora
- Noun phrases
- Verbs
- Nominal premodifiers (e.g. *[tumor] suppressor*), with some additions that we discuss below in the section on domain-specific changes to the guidelines.

**Non-markables** Predicative nouns (e.g. *P53 is [a tumor suppressor gene]*) are not treated as coreferential. There is a separate relation for appositives; markables for the appositive relation are the same as the markables for the identity relation.

Note that singletons (noun phrases, events, etc. (as listed above) that are not in an identity or appositive relation) are not explicitly marked as part of the coreference annotation per se. However, they can be recovered from the syntactic annotation (which was released in Version 1.0 of the CRAFT corpus, but was not available at the time of the coreference annotation), if one wants to take them into account in scoring. (Most coreference resolution scoring metrics ignore singletons, but not all).

#### Piloting the OntoNotes coreference annotation guidelines

After reviewing the pre-existing guidelines, senior annotators marked up a sample full-text article, following the OntoNotes guidelines. The results suggested that the OntoNotes guidelines are a good match to a consensus conception of how coreference should be annotated. Furthermore, the OntoNotes guidelines have been piloted by others, and the project has responded to a number of critiques of earlier guidelines. For example, compared to the MUC-7 guidelines, the treatment of appositives in terms of heads and attributes rather than separate mentions is an improvement in terms of referential status, as is the handling of predicative nouns. The inclusion of verbs and events is a desirable increase in scope. The guidelines are more detailed than in attempts prior to their use in the CRAFT corpus, as well.

#### Domain-specific changes to the OntoNote guidelines

The nature of the biomedical domain required one major adaptation of the guidelines.

**Generics** The OntoNotes guidelines make crucial reference to a category of nominal that they refer to as a generic. (The usage is typical in linguistics, where *generic* refers to a class of things, rather than a specific member of the class [6], e.g. *[Activation loops in protein kinases] are known for their central role in kinase regulation and in the binding of kinase drugs*). Generics in the OntoNotes guidelines include:

- bare plurals
- indefinite noun phrases (e.g. *an oncogene, some teratogens*)
- abstract and underspecified nouns

The status of generics in the OntoNotes annotation guidelines is that they cannot be linked to each other via the IDENTITY relation. They can be linked with subsequent non-generics, but never to each other, so every generic starts a new IDENTITY chain (assuming that it does corefer with subsequent markables).

The notion of a generic is problematic in the biomedical domain. The reason for this is that the referent of any referring expression in a biomedical text is or should be a member of some biomedical ontology, be it in the set of Open Biomedical Ontologies, the Unified Medical Language System, or some nascent or not-yet-extant ontology [86–89]. As such, the referring expression has the status of a named entity. To take an example from BBN, consider the status of *cataract surgery* in the following:

Allergan Inc. said it received approval to sell the PhacoFlex intraocular lens, the first foldable silicone lens available for cataract surgery. The lens’ foldability enables it to be inserted in smaller incisions than are now possible for cataract surgery.

According to the OntoNotes guidelines, *cataract surgery* is a generic, by virtue of being abstract or underspecified, and therefore the two noun phrases are not linked to each other via the IDENTITY relation. However, *cataract surgery* is a concept within the Unified Medical Language System (Concept Unique Identifier C1705869), where it occurs as part of the SNOMED Clinical Terms. As such, it is a named entity like any other biomedical ontology concept, and should not be considered generic. Indeed, it is easy to find examples of sentences in the biomedical literature in which we would want to extract information about the term *cataract surgery* when it occurs in contexts in which the OntoNotes guidelines would consider it generic:

- *Intravitreal administration of 1.25 mg bevacizumab at the time of cataract surgery was safe and effective in preventing the progression of DR and diabetic maculopathy in patients with cataract and DR.* (PMID 19101420)
- *Acute Endophthalmitis After Cataract Surgery: 250 Consecutive Cases Treated at a Tertiary Referral Center in the Netherlands.* (PMID 20053391)
- *TRO can present shortly after cataract surgery and lead to serious vision threatening complications.* (*TRO* is thyroid-related orbitopathy; PMID 19929665).

In these examples, we might want to extract an IS ASSOCIATED WITH relation between <bevacizumab, cataract surgery >, <acute endophthalmitis, cataract surgery >, and <thyroid-related orbitopathy, cataract surgery >. This makes it important to be able to resolve coreference with those noun phrases.

Thus, the CRAFT guidelines differ from OntoNotes in considering all entities to be named entities, so there are no generics in this domain of discourse^1^.

**Prenominal modifiers** A related issue concerned the annotation of prenominal modifiers, i.e. nouns that modify and come before other nouns, such as *cell* in *cell migration*. The OntoNotes guidelines call for prenominal modifiers to be annotated only when they are proper nouns. However, since the CRAFT guidelines considered all entities to be named entities, the CRAFT guidelines called for annotation of prenominal modifiers regardless of whether or not they were proper nouns in the traditional sense.

#### The annotation schema

**Noun groups** The basic unit of annotation in the project is the base noun phrase. (Verbs are also included, as described above in the section on modifiers). The CRAFT guidelines define *base noun phrase* as one or more nouns and any sequence of leftward determiners, adjectives, and conjunctions not separated by a preposition or other noun-phrase-delimiting part of speech; and rightward modifiers such as relative clauses and prepositional phrases. Thus, all of the following would be considered base noun phrases:

- *striatal volume*
- *neural number*
- *striatal volume and neural number*
- *the structure of the basal ganglia*
- *It*

Base noun phrases were not pre-annotated—the annotators selected their spans themselves. This is a potential source of lack of interannotator agreement [90]. Base noun phrases were annotated only when they participated in one of the two relationships that were targetted. Thus, singletons (non-coreferring noun phrases) were not annotated.

**Definitions of the two relations** The two relations that are annotated in the corpus are the IDENTITY relation and the APPOSITIVE relation. The identity relation holds when two units of annotation refer to the same thing in the world. The appositive annotation holds when two noun phrases are adjacent and not linked by a copula (typically the verb *be*) or some other linking word).

**Details of the annotation schema** More specifically, the annotation schema is defined as: IDENTITY chain An IDENTITY chain is a set of base noun phrases and/or appositives that refer to the same thing in the world. It can contain any number of elements. Base noun phrase Discussed above. APPOSITIVE relation An appositive instance has two elements, a head and a set of attributes. The set of attributes may contain just a single element (the prototypical case). Either the head or the attributes may themselves be appositives. Nonreferential pronoun All nonreferential pronouns (pronouns that do not refer to anything, e.g. *It seems to be the case that…*) are included in this single class.

Thus, an example set of annotations would be:

*All brains analyzed in this study are part of [the Mouse Brain Library]* ***a*** *([MBL]* ***b*** *). [The MBL]* ***c*** *is both a physical and Internet resource.* (PMID 11319941) APPOSITIVE chain The Mouse Brain Library _**a**_, MBL _**b**_ IDENTITY chain Mouse Brain Library _**a**_, The MBL _**c**_

### Training of the annotators

We hired two very different types of annotators—linguistics graduate students, and biologists at varying levels of education and with varying specialties. We hired and trained the biologists and the linguists as a single group. Annotators were given a lecture on the phenomenon of coreference and on how to recognize coreferential and appositive relations, as well as nonreferential pronouns. They were then given a non-domain-specific practice document. Following a separate session on the use of the annotation tool, they were given an actual document to annotate. This document is quite challenging, and exercised all of the necessary annotation skills. We began with paired annotation, then introduced a second document for each annotator to mark up individually. Once annotators moved on to individual training annotation, they met extensively with a senior annotator to discuss questions and review their final annotations.

There were 11 total annotators (one lead/senior annotator, 2 senior annotators, and 8 general annotators) made up of two different populations; linguists and biologists. The lead annotator and annotation manager graduated with her M.A. in linguistics and had extensive linguistic annotation and adjudication experience. There were 2 senior annotators other than the lead annotator and who provided annotation for the duration of the project; a linguistics graduate student with several years of linguistic annotation experience and an upper level undergraduate pre-med student with general knowledge in biology, microbiology, physiology, anatomy, and genetics. They contributed about 50% of the single and double annotation efforts overall. The rest of the annotator population was made up of 4 upper level undergraduate biology students, 1 recently graduated linguistics student and 3 linguistics graduate students who were hired and trained at various times throughout the project. All annotators were fully trained at least 6 months before the annotation of data was completed. Prior to hiring, the biology annotators were required to perform a biomedical concept identification task and to demonstrate an understanding of biomedical concepts as evidenced by college transcripts, resumes, and references and upon hiring were trained on basic linguistic concepts and annotation methods. The linguists were required to have previous linguistic annotation experience and prior to hiring performed a biomedical terminology noun phrase identification task. Each was required to demonstrate their linguistics background via resumes and references. These 8 annotators collectively contributed the other 50% of single and double annotation efforts.

During the initial training phase, we paired biologists with linguists and had them work on the same article independently, then compare results. This turned out to be an unnecessary step, and we soon switched to having annotators work independently from the beginning.

### Two populations of annotators

Impressionistically, we did not notice any difference in their performance. The biologists were able to grasp the concept of coreference, and the linguists did not find their lack of domain knowledge to be an obstacle to annotation. This accords with [91]’s observation that expertise in an annotation task can be an entirely different question from expertise in linguistics or expertise in a domain—both groups seemed to exhibit similar abilities to do the annotation task.

### The annotation process

There are no ethical oversight *requirements* related to corpus construction. We voluntarily reviewed the project in light of the Ethical Charter on Big Data (which includes linguistic corpus preparation) [92] and identified no issues.

Most articles in coreference layer of the CRAFT corpus are single-annotated. A subset of ten articles was double-annotated by random pairs of annotators in order to calculate inter-annotator agreement.

The length of the articles means that a single IDENTITY chain can extend over an exceptionally long distance. The median length was two base noun phrases, but the longest was 186 (Table 7). To cope with this, annotators typically marked up single paragraphs as a whole, and then linked entities in that paragraph to earlier mentions in the document. In the case of questions, annotators had access to senior annotators via email and meetings. Annotation was done using Knowtator, a Protégé plug-in (Ogren, 2006a; Ogren, 2006b).

**Table 7:** Descriptive statistics of coreference annotations in the CRAFT corpus

IDENTITY chains	23,887
APPOSITIVE	4591
Pronouns	4145
Mean IDENT chains per paper	246.3
Median IDENT chains per paper	236
Mean APPOS per paper	47.3
Median APPOS per paper	43
Mean length of IDENT chains	4
Median length of IDENT chains	2
Longest IDENT chain	186
Within-sentence IDENT chains	1495
Between-sentence IDENT chains	22,392

### Calculation of inter-annotator agreement

The inter-annotator agreement gives some indication of the difficulty of the annotation task and the consistency of annotations, and also suggests an upper bound for the performance of automatic techniques for coreference resolution on this data [93, 94]. Inter-annotator agreement was calculated using the code described in [95]. Average inter-annotator agreement over a set of ten articles is 0.684 by the MUC metric. We give a number of other metrics in Table 3 (MUC, [96], B3, [97], CEAF, [98], and Krippendorff’s alpha [99, 100]). We note that the value for Krippendorff’s alpha is lower than the 0.67 that Krippendorff indicates must be obtained before values can be conclusive, but no other inter-annotator agreement values for projects using the OntoNotes guidelines have been published to which to compare these numbers.

### Benchmarking methodology

To assess the difficulty of the task of resolving the coreference relationships in this data, we ran three experiments using two different coreference resolution systems and an ensemble system. One is a publicly available coreference resolution system. It is widely used and produces at- or near-state-of-the-art results on newswire text. It uses a rule-based approach. (We do not name the system here because the results are quite low, and we do not want to punish the authors of this otherwise high-performing system for making their work freely publicly available). The other is a simple rule-based approach that we built with attention to some of the specifics of the domain. (We do not go into detail about the system as it will be described in a separate publication). To do the benchmarking, we ran the publicly available system with its default parameters. (Since it is a rule-based system, this affected only the preprocessing steps, not the actual coreference resolution).

The output of both systems was scored with the CoNLL scoring script [37]. We encountered a number of difficulties at both stages of the process. The Simple system outputs pairs, but the CRAFT IDENTITY chains can be arbitrarily long. This is a general issue that is likely to occur with many coreference resolution systems that assume the mention pair model [101] without subsequent merging of pairs. For evaluation purposes, the pairs that are output by Simple were mapped to any corresponding IDENTITY or APPOSITIVE chain as part of the scoring process. A mention pair is scored as correct if both the anaphor and the antecedent appear in the corresponding chain.

Because ensemble systems have proven to be quite useful for many language processing tasks [102–105], we also unioned the output of the two systems.

## Results

### Descriptive statistics of annotations

Descriptive statistics of the annotations are given in Table 7. As can be seen, the IDENTITY and APPOSITIVE chains add up to over 28,000 annotations.

### Benchmarking results

We compare performance of each coreference resolution system, as well as the combined result of these two systems, in Table 9. The evaluation combines performance on the IDENTITY and APPOSITIVE relations, since it is the combination of these that constitutes coreference in CRAFT. The publicly available system is referred to as System A, and the domain-adapted simple rule-based system is referred to as Simple.

Both systems achieved considerably higher precision than recall, which is not surprising for rule-based systems. Overall, the domain-adapted Simple system considerably outperformed the general-domain System A. The ensemble system had slightly improved performance, with unchanged precision, but slightly improved recall. All output from the scoring script is available on the associated SourceForge site.

## Discussion

The data that is present in the CRAFT corpus coreference annotations should be useful to linguists researching coreferential phenomena and to natural language processing researchers working on on coreference resolution. Can it have an impact beyond that? We analyzed the overlap between the IDENTITY chains in CRAFT and the named entity annotation in CRAFT. The motivation for assessing the extent of this overlap is that any IDENTITY chain that can be resolved to a named entity is a possible input to an information extraction algorithm that targets that type of entity. The analysis showed that 106,263 additional named entities can be recovered by following the IDENTITY chains in the full 97-paper corpus. This represents an increase of 76% in the possible yield of information extraction algorithms; if that proportion holds across other corpora, the potential value of text mining of the scientific literature would increase considerably.

Reflecting on this project, what we learnt suggests two changes we might have made to our approach. First, we could have pre-annotated all of the base noun phrases; doing so can increase inter-annotator agreement in coreference annotation [90]. Second, we could have marked generics (adhering to the OntoNotes guidelines), while allowing them to be linked to each other by IDENTITY relations; doing so would have allowed a simple programmatic transformation to modify our corpus so that it was completely consonant with the OntoNotes guidelines.

With respect to questions of reproducibility and where this work is positioned in relation to previous work on coreference, we note that the benchmarking results demonstrate a dramatic decrease in performance of systems that work well on newswire text. The inter-annotator agreement numbers in Table 8 suggest that the annotation is consistent, and those inter-annotator agreement values are far higher than the performance numbers in Table 9. The most likely explanation for the poor performance of existing systems is that automated coreference resolution is more difficult in biomedical journal articles than newswire text, or, at the very least, that systems tuned for newswire text need significant alteration to perform as well in biomedical journal articles. One possible factor in this difficulty is much greater length of the documents and, consequently, much longer coreference chains. Certainly the low performance of both of the baseline systems cannot be blamed on inconsistencies in training data, since both of the baseline systems are rule-based and neither gets trained.

**Table 8:** Inter-annotator agreement

Metric	Average
MUC	0.684
Class-B3	0.858
Entity-B3	0.750
Mention-based CEAF	0.644
Entity-based CEAF	0.480
Krippendorff’s alpha	0.619

**Table 9:** Benchmarking results: System A, Simple, and the union of the two

	B3			BLANC		
System	P	R	F	P	R	F
System A	0.93	0.08	0.14	0.93	0.026	0.05
Simple	0.78	0.29	0.42	0.78	0.22	0.33
Union	0.78	0.35	0.46	0.78	0.26	0.37

## Conclusions

The CRAFT coreference corpus is an improvement over related projects in a number of ways, particularly the unrestricted definition of markable, connection to extensive annotation of semantic classes (without restriction to those classes), and connection with complete phrase structure annotation. We hope that these qualitative differences from prior coreference annotation work in the biomedical domain will be a contribution to the effort to bring the performance of coreference resolution tools in the domain to the level that is seen in newswire text.

## Endnote

^1^ Note that in our guidelines, as in the OntoNotes project, indefinite noun phrases are used to start new IDENTITY chains, and are not linked with previous markables, but this is because they are discourse-new, not because we consider them to be generics.

## Abbreviations

BCE: Before the common Era (a reference point for calendar dates)
BioNLP-ST: BioNLP shared task (a shared task in biomedical natural language processing)
CEAF: Constrained EntityAlignment F-Measure (a metric for evaluating coreference resolution systems)
CoNLL: Conference on natural language learning
CRAFT: Colorado richly annotated full text (a corpus of full-text journal articles about mouse genomics)
LLL: Learning language in logic (a shared task in biomedical natural language processing)
MUC-6: Message understanding conference 6 (an influential shared task in natural language processing)
MUC-7: Message understanding conference 7 (an influential shared task in natural language processing)
RTE: Recognizing textual entailment (a task definition in natural language processing)
XML: Extensible markup language
